# Identification and diagnostic potential of serum microRNAs as biomarkers for early detection of Alzheimer’s disease

**DOI:** 10.18632/aging.205165

**Published:** 2023-11-01

**Authors:** Ying-Hao Han, Hong-Yi Xiang, Dong Hun Lee, Lin Feng, Hu-Nan Sun, Mei-Hua Jin, Taeho Kwon

**Affiliations:** 1 College of Life Science and Biotechnology, Heilongjiang Bayi Agricultural University, Daqing, P.R. China; 2 Department of Biological Sciences, Research Center of Ecomimetics, Chonnam National University, Gwangju, Republic of Korea; 3 Primate Resources Center, Korea Research Institute of Bioscience and Biotechnology (KRIBB), Jeongeup, Jeonbuk, Republic of Korea; 4 Department of Functional Genomics, KRIBB School of Bioscience, University of Science and Technology, Daejeon, Republic of Korea

**Keywords:** Alzheimer’s disease, serum, microRNA, diagnostic models, biomarkers

## Abstract

This study aimed to investigate the differential expression of serum microRNAs in cognitive normal subjects (NC), patients with mild cognitive impairment (MCI), and patients with Alzheimer’s disease (AD), with the objective of identifying potential diagnostic biomarkers. A total of 320 clinical samples, including 32 MCI patients, 288 AD patients, and 288 healthy controls, were collected following international standards. The expression of microRNAs in serum was analyzed using the Agilent human microRNA oligonucleotide microarray, and bioinformatics methods were employed to predict target genes and their involvement in AD-related pathways. Among the 122 microRNAs screened, five microRNAs (hsa-miR-208a-5p, hsa-miR-125b-1-3p, hsa-miR-3194-3p, hsa-miR-4652-5p, and hsa-miR-4419a) exhibited differential expression and met quality control standards. Bioinformatics analysis revealed that the target genes of these microRNAs were involved in multiple AD-related pathways, which changed with disease progression. These findings demonstrate significant differences in serum microRNA expression between NC, MCI, and AD patients. Three microRNAs were identified as potential candidates for the development of diagnostic models for MCI and AD. The results highlight the crucial role of microRNAs in the pathogenesis of AD and provide a foundation for the development of novel therapeutic strategies and personalized treatment approaches for AD. This study contributes to the understanding of AD at the molecular level and offers potential avenues for early diagnosis and intervention in AD patients.

## INTRODUCTION

Alzheimer’s disease (AD) is a complex, heterogeneous, and progressive disease that is the most common type of neurodegenerative dementia [1]. The pathogenesis of AD is regulated by many factors, which involve complex molecular mechanisms, leading to the degeneration of neurons [2]. At present, with the aging of the population, the number of AD cases is expected to continue to increase, placing a heavy burden on the healthcare systems of various countries. There is no drug to cure Alzheimer’s disease, and most patients with Alzheimer’s disease are in the final stage when they are discovered, and treatment is difficult. Therefore, the development of early non-invasive diagnostic methods and the identification of novel biomarkers are urgently needed new diagnostic methods capable of detecting highly specific biomarkers at relatively low cost in the early stages of Alzheimer’s disease [3–5]. With the development of modern medical diagnostic analysis, it has become possible to accurately detect highly specific molecules and identify several biomarkers of AD, including genes and proteins responsible for Alzheimer’s disease, markers of neuronal apoptosis, markers of inflammation in the blood, and markers of synaptic dysplasia [6]. Currently, miRNAs are widely recognized as potential biomarkers due to their stable expression and high availability in body fluids such as cerebrospinal fluid, breast milk, urine, blood, seminal plasma, and tears [7]. Previous studies have shown miRNAs have responses in the pathological development of neurodegenerative diseases, including oxidative stress, neuroinflammation, protein aggregation, and changes in neuronal development and plasticity, suggesting that molecular pathways regulated by miRNAs may regulate the progression of neurodegenerative diseases at an early stage [8]. Meanwhile, studies on the transformation of MCI to AD have found that miR-34c is up-regulated in the hippocampus of Alzheimer’s model mice, and the expression of miR-30a-5p is significantly increased in AD patients [9, 10]. Therefore, miRNA is a more sensitive biomarker for early AD detection. The use of microRNA (miRNA) as markers of AD has considerable potential, and diagnostic studies based on miRNA groups have shown that AD has the potential to diagnose individual patients with high accuracy.

MicroRNAs (miRNAs) are a class of small non-coding RNAs of eukaryotic cells with a length of about 22 nucleotides, widely present in all tissues and body fluids of human body, such as blood, urine, saliva, milk and cerebrospinal fluid [11]. In body fluids, miRNAs often exist in the form of complexes with proteins, etc., which resist the degradation of RNA enzymes and are highly stable and conserved, playing a key regulatory role in the body’s pathophysiological process [12]. The expression profile of miRNA is consistent among healthy individuals, but it can change significantly under different disease states. The change of the expression profile is often earlier than the biochemical and imaging changes, which is an ideal potential marker for early diagnosis of disease, The collection of miRNAs in serum samples is relatively easy and can be obtained multiple times in a non-invasive manner, facilitating long-term monitoring [13]. With the further understanding of miRNA and the optimization of functional analysis techniques of miRNA, the role of miRNA in various physiological processes has been further explained, and the change of miRNA expression profile in various diseases has gradually been paid attention to. In the early stage, scholars mainly focused on the application of miRNA in plasma in diseases. Due to the existence of blood-brain barrier, the entry of central-specific miRNA into blood is restricted. In recent years, the research on the mechanism of action of miRNA in cerebrospinal fluid in central nervous system diseases has been gradually paid attention to [14]. Previous studies have found that the expression level of miR-29a in the cerebrospinal fluid of AD patients is lower than that of the control group, and the decrease of miR-29a can up-regulate the activity of BACE1, induce the production of Aβ protein, lead to excessive deposition of Aβ in the brain tissue, and form the specific pathological markers of AD. Due to the regulatory effect of miR-29a on the production of Aβ protein, it is speculated that the detection of miRNA in cerebrospinal fluid may be A potential biomarker or therapeutic target for the diagnosis of AD [15]. Muller et al. have confirmed the diagnostic value of cerebrospinal fluid miR-29a, with a sensitivity of 89% and a specificity of 70% in the diagnosis of AD [16]. In addition, it was found that the expression level of miR-26b in the cerebrospinal fluid of AD patients was significantly increased, and the up-regulation of miR-26b increased tau phosphorylation and neuronal cell apoptosis, promoting the occurrence and development of AD [17]. Most importantly, miRNA expression profile changes in pathological state are earlier than biochemical and other imaging changes, which has special significance for the early diagnosis of AD. For example, when there is only mild cognitive impairment at the earliest stage of AD, no corresponding changes in Aβ can be detected. At this time, the expression of miR-9, miR-125b, miR-146b and miR-155 in cerebrospinal fluid is significantly up-regulated, suggesting that miRNA is of early diagnostic value for AD [18]. In addition, miRNAs also show high application value in the differential diagnosis of diseases. A recent meta-analysis showed that cerebrospinal fluid Aβ and tau proteins alone were limited in the differential diagnosis of AD and other types of dementia (vascular dementia, frontotemporal dementia, and Lewy bodies dementia), with low sensitivity (70%-75%) and specificity (65%-80%). However, the use of the ratio of miR-29c-3p/miR-15a-5p in the differential diagnosis of these two diseases can achieve high efficacy [14, 19]. In summary, in-depth study of miRNA expression profile changes in cerebrospinal fluid of AD patients will help clarify the role of miRNA in the pathogenesis of AD, provide a new strategy for the diagnosis and treatment of AD, and have a broad application prospect.

Therefore, this study will explore key regulatory pathways in the development of Alzheimer’s disease based on data sets such as miRNA expression profile analysis in healthy control samples, mild cognitive impairment samples and Alzheimer’s samples. It was found that hsa-miR-208a-5p, hsa-miR-125b-1-3p, hsa-miR-3194-3p, hsa-miR-4652-5p and hsa-miR-4419a5 miRNA played a role in the regulation. KEGG and GO enrichment analysis showed that they regulate SNARE interactions in vesicular transport, negative regulation of biological processes, cellular component organization or biogenesis and PI3K-AKT signaling pathways, respectively, mediating the development process of Alzheimer’s disease.

## RESULTS

### Normalization of data and differential gene analysis

**Figure 1:**
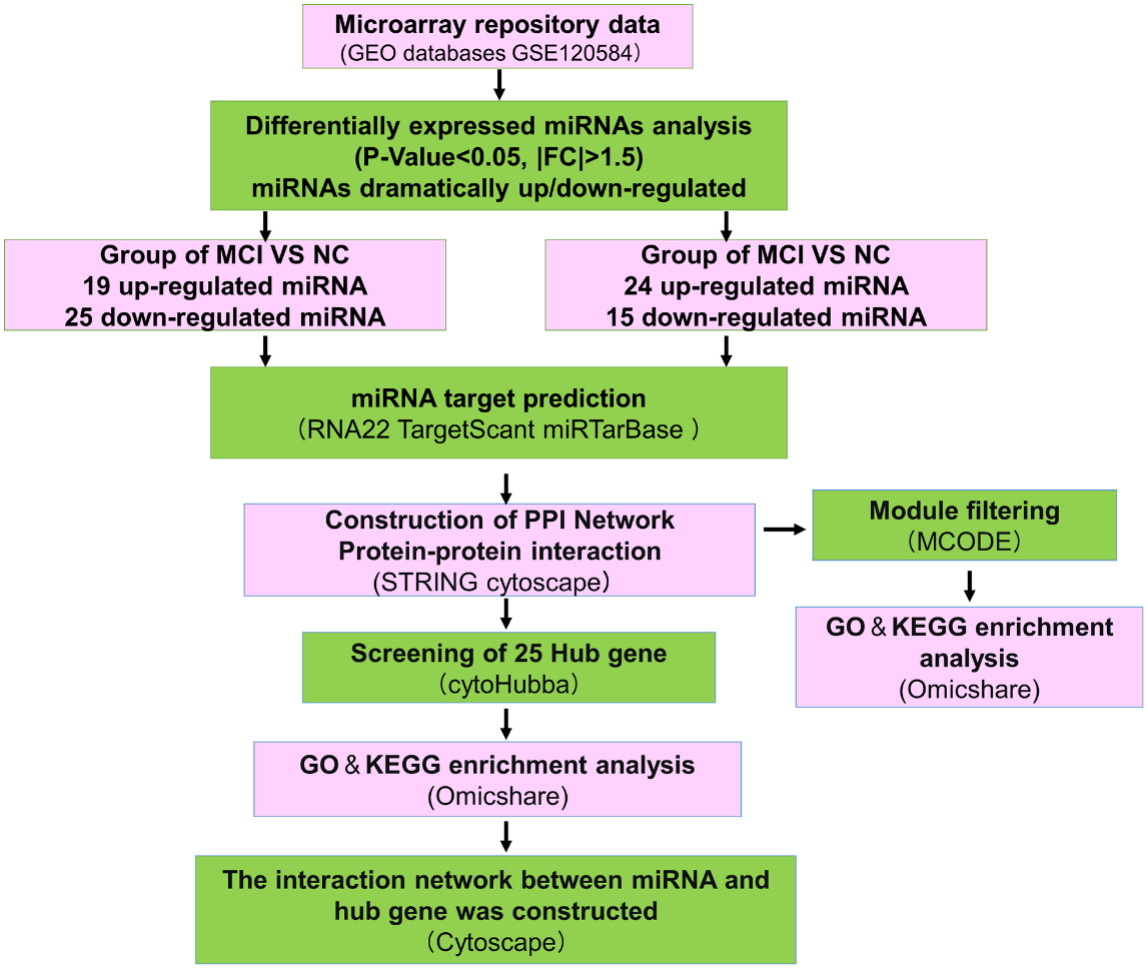
**Overall working flowchart of bioinformatics analysis based on publicly available data in GEO public database.** GEO, Gene Expression Omnibus; miRNA, microRNA; FC, fold change; RNA22, TargetScant, miRTarBase, microRNA-target interactions database; mRNA, messenger RNA. NC, normal control; MCI, Mild cognitive impairment; AD, Alzheimer’s disease.

**Figure 2:**
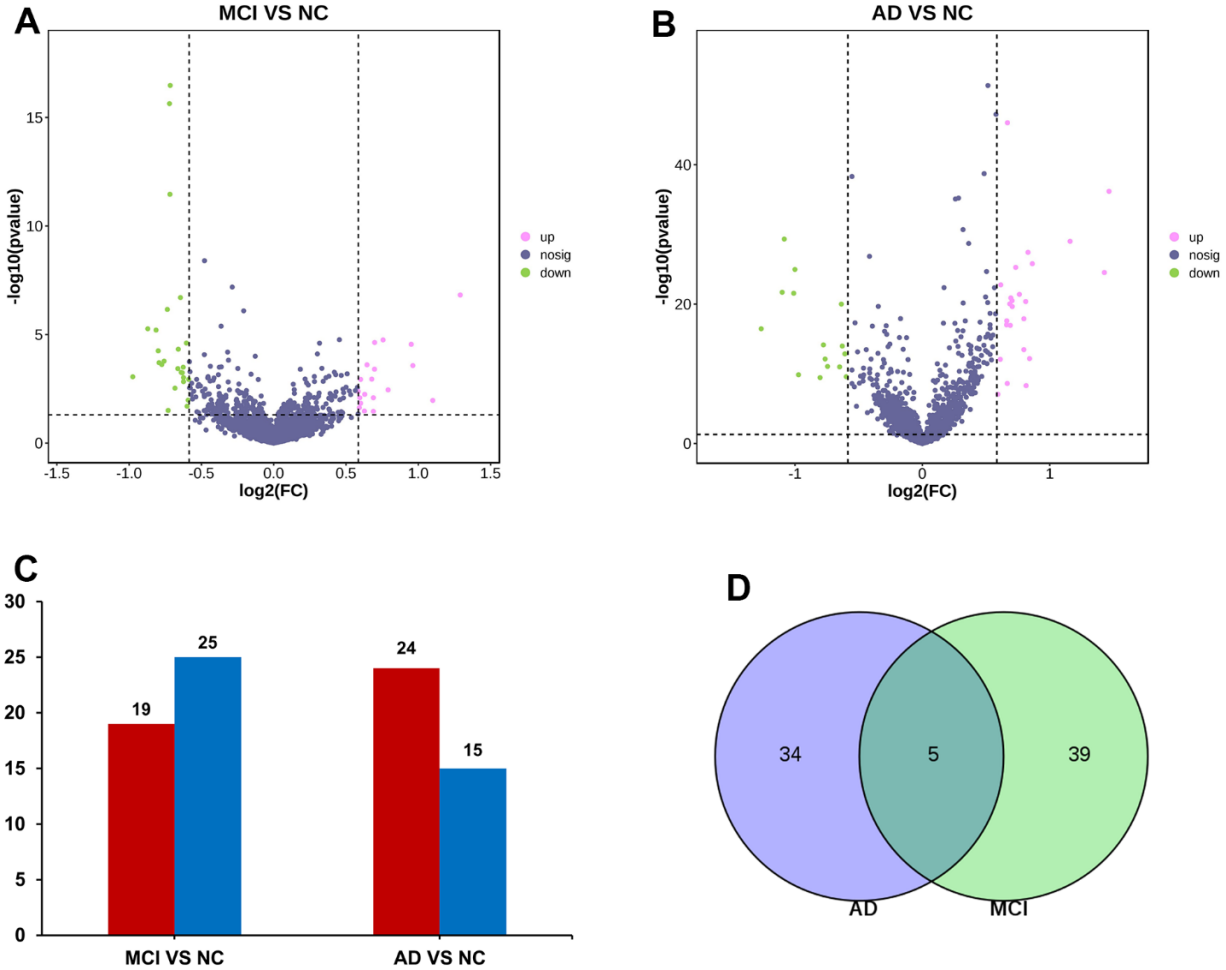
**miRNA screening.** (**A**) Volcano maps of miRNA differentially expressed in MCI. Red dots indicate up-regulated genes, P < 0.05, FC > 1.5; Green dots indicate down-regulated genes, P < 0.05, FC < -1.5; Black dots indicate genes with no significant difference in expression. (**B**) Volcano maps of miRNA differentially expressed in AD. (**C**) Differential miRNA data statistics. (**D**) Venn diagram screened miRNAs that were differentially expressed in both MCI and AD groups.

The data of NC, MCI, and AD in GSE120584 were analyzed differentially using GEO2R to obtain miRNAs differentially expressed in MCI and AD, and then the differential miRNAs were screened with p-value ≤ 0.05, Fold change ≥ 1.5 or Fold change ≤ -1.5 to obtain up-regulated miRNAs and down-regulated (Figure 1). The results of miRNAs in which there were 19 up-regulated miRNA and 25 down-regulated miRNA in MCI VS NC group (Figure 2A). There were 24 up-regulated miRNA expression and 15 down-regulated miRNA expression in AD VS NC group (Figure 2B); The columnar statistics of the different miRNAs were performed, red indicates upward adjustment and blue indicates downward adjustment (Figure 2C). Then the miRNAs that were co-differentially expressed in both MCI and AD were obtained by using Venn diagram analysis (Figure 2D), and a total of 5 differential miRNAs (hsa-miR-208a-5p, hsa-miR-125b-1-3p, hsa-miR-3194-3p, hsa-miR-4419a, hsa-miR-4652-5p) were obtained (Table 1).

**Table 1:** Five differentially expressed miRNAs at intersection.

**Name**	**MCI VS NC**		**AD VS NC**	
	**log_2_FC**	**P-value**	**log_2_FC**	**P-value**
hsa-miR-125b-1-3p	-0.73	3.13E-02	1.4287336	2.95E-25
hsa-miR-3194-3p	1.1	1.08E-02	-1.2653072	3.47E-17
hsa-miR-4652-5p	1.29	1.50E-07	-1.0004974	1.07E-25
hsa-miR-4419a	-0.793	1.98E-04	0.7057729	2.21E-20
hsa-miR-208a-5p	0.645	2.48E-04	-1.0843337	4.70E-30

### Target gene prediction for predicting differential miRNAs

**Figure 3:**
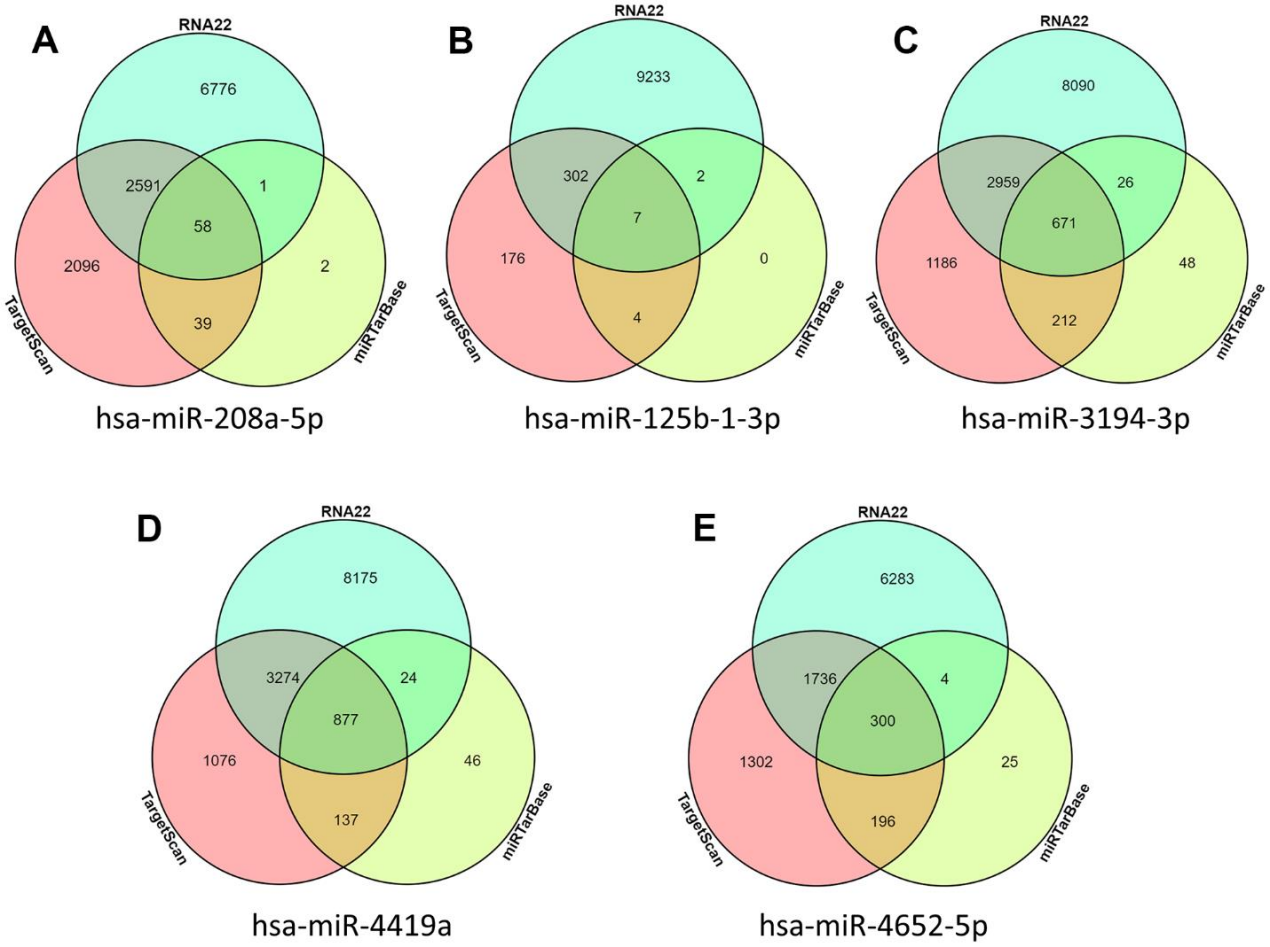
**miRNA target gene prediction.** (**A**) hsa-miR-208a-5p predicted target gene; (**B**) hsa-miR-125b-1-3p predicted target gene; (**C**) hsa-miR-3194-3p predicted target gene; (**D**) hsa-miR-4419a predicted target gene; (**E**) hsa-miR-4652-5p predicted target gene.

In order to explore how these intersection differences of miRNA play a role in the occurrence and development of AD, we used miRNA prediction tools miRTarBase, RNA22, and TarsCant to predict what target genes these miRNAs can control. Finally, according to the Venn diagram, the prediction target genes from three websites were interexchange. Intersection target genes predicted by the three sites simultaneously were obtained. Among them, hsa-miR-208a-5p predicted a total of 58 target genes (Figure 3A), hsa-miR-125b-1-3p predicted a total of 7 target genes (Figure 3B), hsa-miR-3194-3p a total of 671 target genes were predicted (Figure 3C), hsa-miR-4419a a total of 877 target genes were predicted (Figure 3D), hsa-miR-4652-5p a total of 300 target genes were predicted (Figure 3E).

### PPI network construction

**Figure 4:**
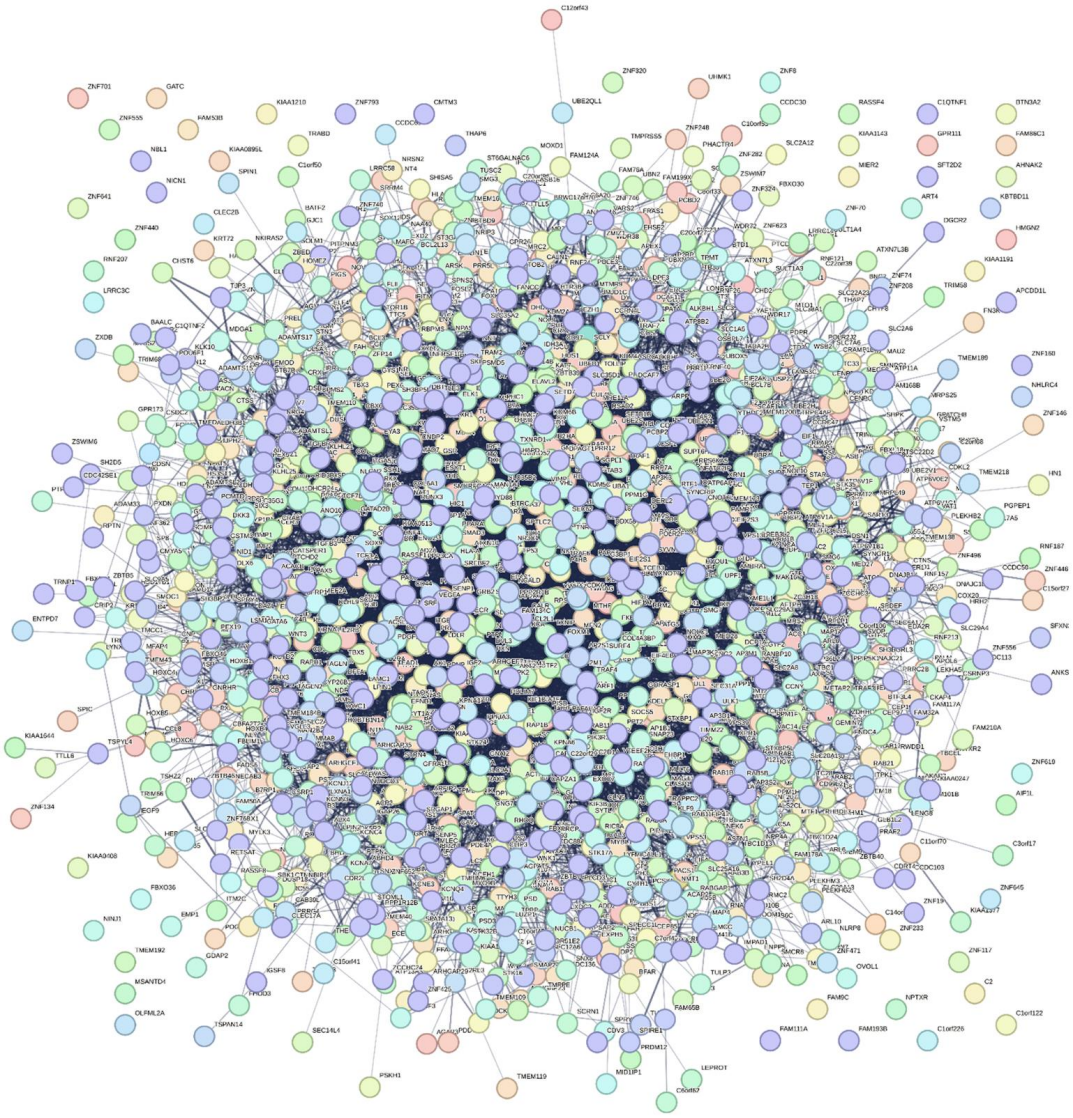
Five differential miRNAs (hsa-miR-208a-5p, hsa-miR-125b-1-3p, hsa-miR-3194-3p, hsa-miR-4419a, hsa-miR-4652-5p) PPI network constructed by STRING.

In order to explore how target genes play a role in the occurrence and development of AD disease, PPI network interaction was performed on all genes targeted by 5 miRNAs using STRING website (Figure 4A). The regulatory network of target gene interaction was obtained for subsequent analysis.

### Module screening of PPI network

**Figure 5:**
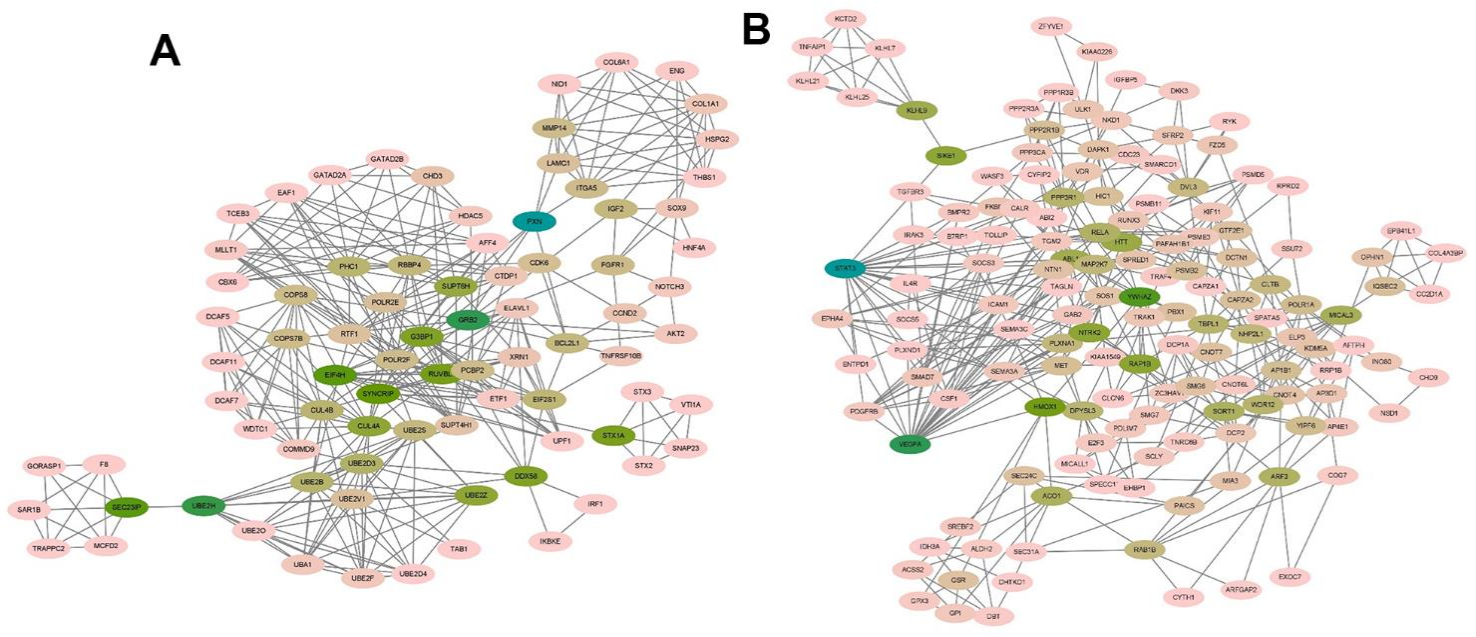
**The module identified from the PPI network using the MCODE method (1).** (**A**) Module 1 with an MCODE score of 8.321. (**B**) Shows module 2 with an MCODE score of 7.868.

**Figure 6:**
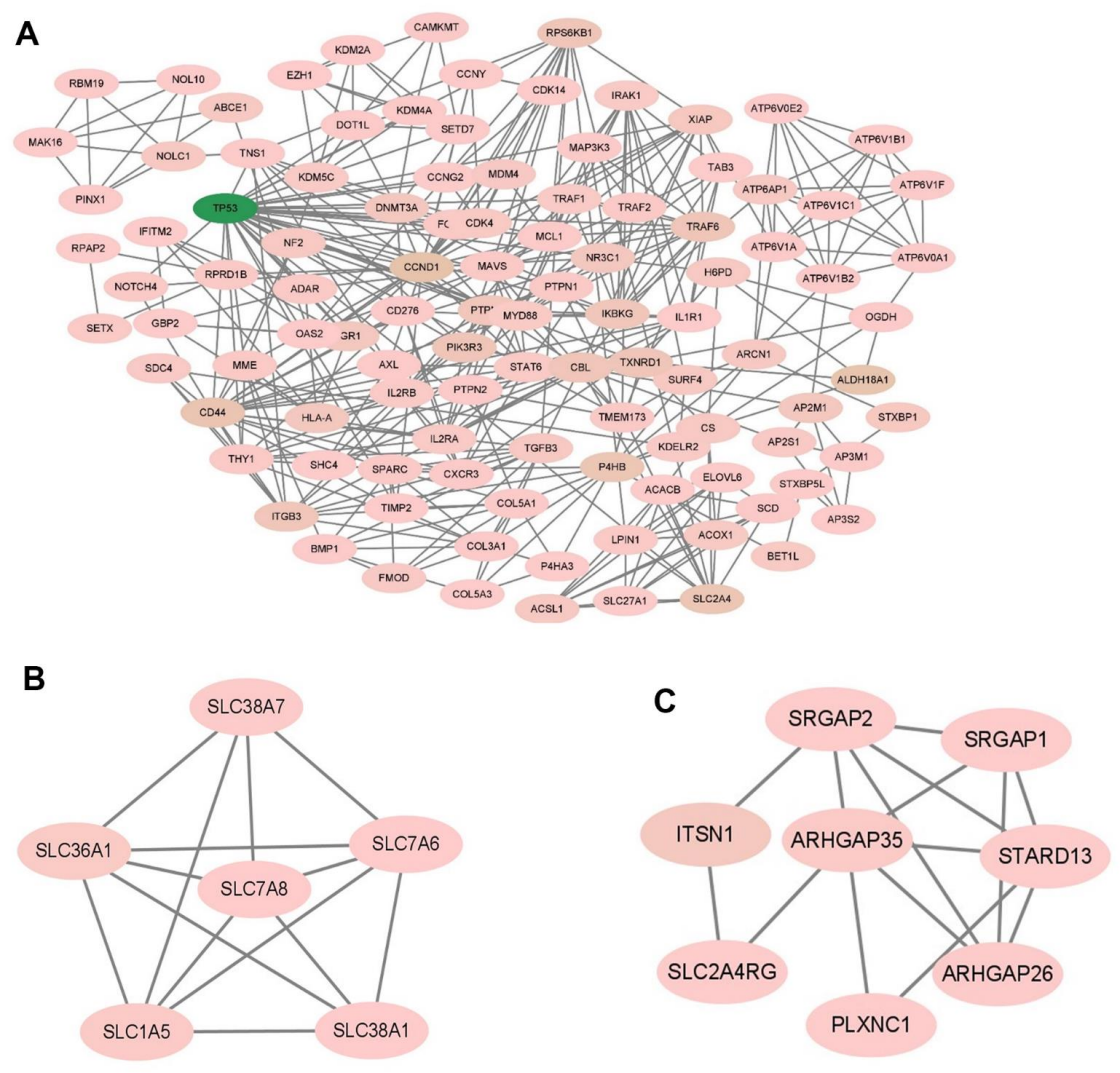
**The module identified from the PPI network using the MCODE method (2).** (**A**) The module 3 with an MCODE score of 5.764. (**B**) The module 4 with an MCODE score of 5.600. (**C**) The module 5 with an MCODE score of 4.286. MCODE, molecular complex detection.

The MCODE plug-in in Cytoscape was used to perform module analysis on the constructed protein-protein interaction (PPI) network. A total of 42 modules were identified, and the top 5 modules with the highest scores were selected. Module 1 had 82 nodes and 337 edges, scoring 8.321 (Figure 5A). Module 2 consisted of 107 nodes and 417 edges, with a score of 7.868 (Figure 5B). Module 3 included 145 nodes and 415 edges, scoring 5.764 (Figure 6A). Module 4 contained 6 nodes and 14 edges, scoring 5.600 (Figure 6B). Module 5 comprised 8 nodes and 15 edges, scoring 4.286 (Figure 6C).

### GO enrichment and KEGG analysis of modules with the highest scores

**Figure 7:**
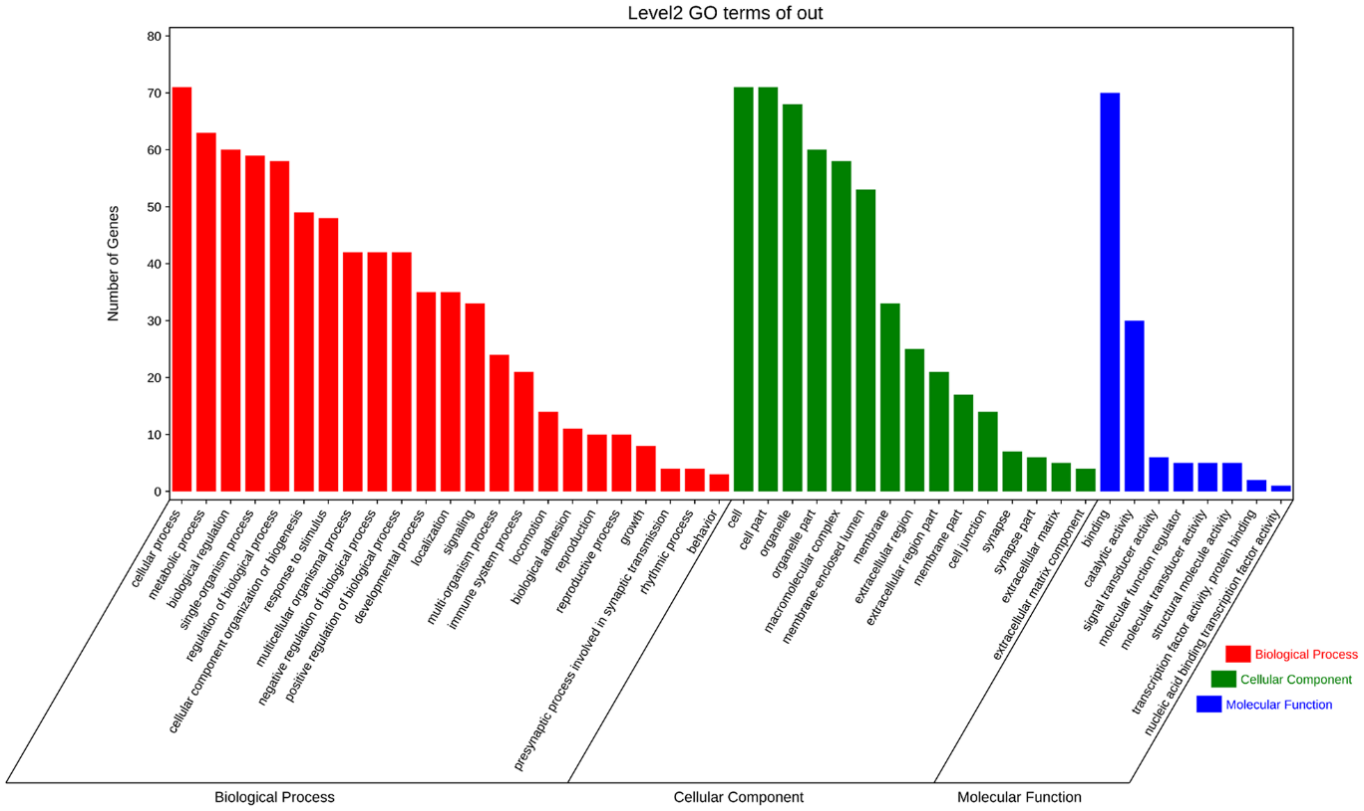
**Enrichment analysis of fundamental functional factors in maximum modules.** GO enrichment analysis of the most significant modules, including biological processes (BP), cell components (CC), molecular functions (MF).

**Figure 8:**
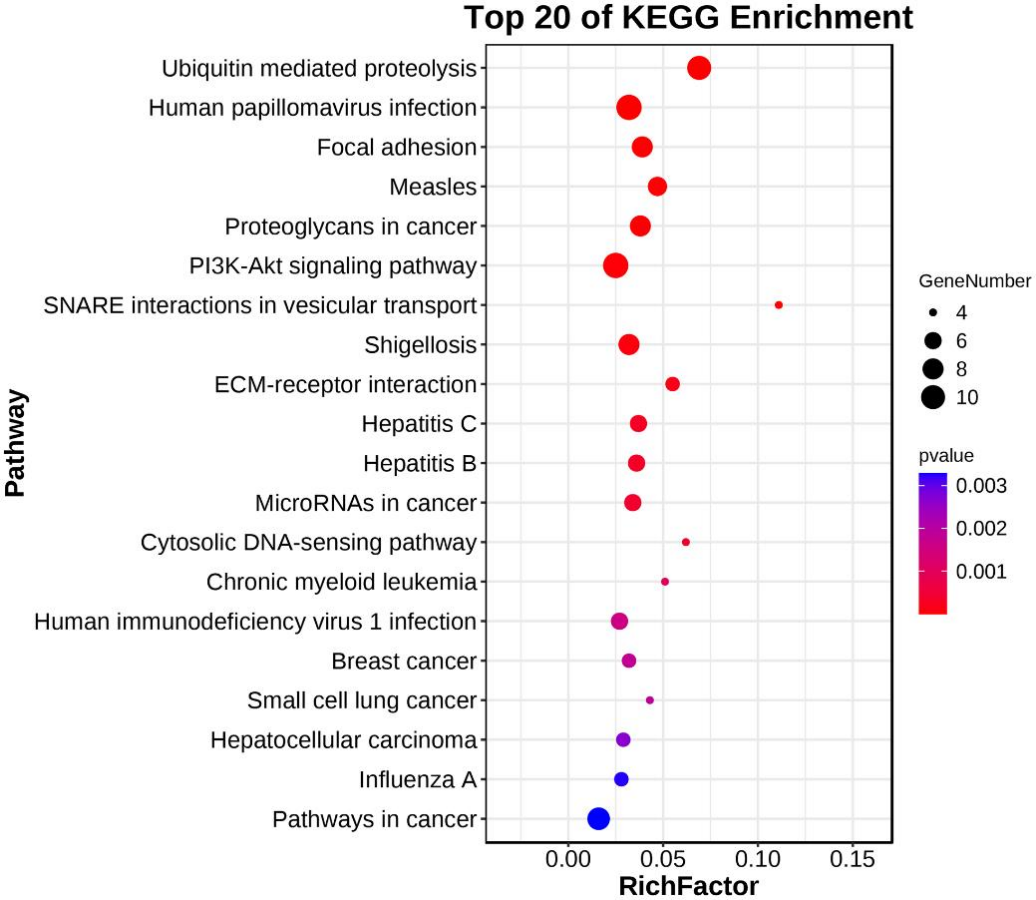
KEGG pathway enrichment analysis demonstrated that the hub gene was enriched in SNARE interactions in vesicular transport and Focal adhesion pathways.

GO analysis and KEGG pathway enrichment were performed on the significant modules using the OmicShare platform to investigate their biological functions. The results indicated that these genes were primarily involved in cellular processes, metabolic processes, and biological regulation according to GO analysis (Figure 7 and Table 2). In terms of cellular components, the genes were mainly associated with organelle parts, macromolecular complexes, and membrane-enclosed lumens. Additionally, the molecular functions of these genes were predominantly related to binding and catalytic activities. KEGG analysis revealed enrichment in SNARE interactions in vesicular transport and Focal adhesion pathways. These findings provide insights into the potential roles and pathways involved in the biological functions of the hub genes in these modules. (Figure 8 and Table 3).

**Table 2:** GO enrichment analysis of the largest module.

**Group**	**GO ID**	**Description**	**Out (71)**	**All (17723)**	**P-value**	**FDR**
Biological processes	GO:0009987	Cellular process	71	16526	0.006908	0.050286
	GO:0008152	Metabolic process	63	12118	5.54E-05	0.001583
	GO:0065007	Biological regulation	60	12377	0.003443	0.031528
	GO:0044699	Single-organism process	59	14826	0.6256	0.673281
	GO:0050789	Regulation of biological process	58	11848	0.004124	0.035405
	GO:0071840	Cellular component organization or biogenesis	49	7131	8.50E-07	7.38E-05
	GO:0050896	Response to stimulus	48	9222	0.005579	0.042975
	GO:0032501	Multicellular organismal process	42	7719	0.005783	0.0441
	GO:0048519	Negative regulation of biological process	42	5491	8.35E-07	7.38E-05
	GO:0048518	Positive regulation of biological process	42	6568	0.000125	0.002867
	GO:0032502	Developmental process	35	6569	0.023237	0.100339
	GO:0051179	Localization	35	6686	0.030489	0.119911
	GO:0023052	Signaling	33	6692	0.082487	0.198103
	GO:0051704	Multi-organism process	24	2820	0.000158	0.003329
	GO:0002376	Immune system process	21	3279	0.015624	0.079108
	GO:0040011	Locomotion	14	2010	0.026859	0.110606
	GO:0022610	Biological adhesion	11	1630	0.058762	0.165351
	GO:0022414	Reproductive process	10	1469	0.067135	0.177013
	GO:0000003	Reproduction	10	1472	0.067873	0.177758
	GO:0040007	Growth	8	945	0.034733	0.129229
	GO:0048511	Rhythmic process	4	343	0.048751	0.153181
	GO:0007610	Behavior	3	792	0.620541	0.670072
Cell components	GO:0005623	Cell	71	17034	0.002134	0.01277
	GO:0044464	Cell part	71	17034	0.002134	0.01277
	GO:0043226	Organelle	68	13845	2.20E-06	3.01E-05
	GO:0044422	Organelle part	60	10271	1.63E-07	3.44E-06
	GO:0032991	Macromolecular complex	58	6212	7.39E-17	2.59E-14
	GO:0031974	Membrane-enclosed lumen	53	5614	1.42E-14	1.15E-12
	GO:0016020	Membrane	33	9701	0.86252	0.897521
	GO:0005576	Extracellular region	25	4295	0.014039	0.047546
	GO:0044421	Extracellular region part	21	3465	0.017102	0.053855
	GO:0044425	Membrane part	17	7099	0.996365	1
	GO:0030054	Cell junction	14	1386	0.000666	0.005194
	GO:0045202	Synapse	7	1227	0.187558	0.291486
	GO:0044456	Synapse part	6	886	0.12243	0.20443
	GO:0031012	Extracellular matrix	5	341	0.009818	0.041855
	GO:0044420	Extracellular matrix component	4	126	0.001382	0.009185
Molecular functions	GO:0005488	Binding	70	16807	0.012356	0.06197
	GO:0003824	Catalytic activity	30	5845	0.040288	0.113998
	GO:0004871	Signal transducer activity	6	1688	0.643943	0.724983
	GO:0005198	Structural molecule activity	5	727	0.14896	0.235913
	GO:0098772	Molecular function regulator	5	1450	0.667532	0.74395
	GO:0060089	Molecular transducer activity	5	1667	0.782385	0.851873
	GO:0000988	Transcription factor activity, protein binding	2	522	0.602276	0.685063
	GO:0001071	Nucleic acid binding transcription factor activity	1	1416	0.996644	0.999664

**Table 3:** KEGG enrichment analysis of the largest module.

**ID**	**Description**	**Num**	**Per**	**P-value**	**Q-value**
ko04120	Ubiquitin mediated proteolysis	10	21.277	5.40E-09	8.16E-07
ko05165	Human papillomavirus infection	11	23.404	2.55E-06	1.92E-04
ko04510	Focal adhesion	8	17.021	1.66E-05	6.40E-04
ko05162	Measles	7	14.894	1.88E-05	6.40E-04
ko05205	Proteoglycans in cancer	8	17.021	2.12E-05	6.40E-04
ko04151	PI3K-Akt signaling pathway	11	23.404	2.80E-05	7.04E-04
ko04130	SNARE interactions in vesicular transport	4	8.511	4.66E-05	1.00E-03
ko05131	Shigellosis	8	17.021	6.88E-05	1.30E-03
ko04512	ECM-receptor interaction	5	10.638	0.0001513	2.54E-03
ko05160	Hepatitis C	6	12.766	0.0002885	4.36E-03
ko05161	Hepatitis B	6	12.766	0.000328785	4.51E-03
ko05206	MicroRNAs in cancer	6	12.766	0.000435621	5.19E-03
ko04623	Cytosolic DNA-sensing pathway	4	8.511	0.000447226	5.19E-03
ko05220	Chronic myeloid leukemia	4	8.511	0.000994005	1.07E-02
ko05170	Human immunodeficiency virus 1 infection	6	12.766	0.001573508	1.58E-02
ko05224	Breast cancer	5	10.638	0.001785237	1.68E-02
ko05222	Small cell lung cancer	4	8.511	0.001896346	1.68E-02
ko05225	Hepatocellular carcinoma	5	10.638	0.002667643	2.24E-02
ko05164	Influenza A	5	10.638	0.00325053	2.48E-02
ko05200	Pathways in cancer	9	19.149	0.003290298	2.48E-02

### Screening of hub genes

**Figure 9:**
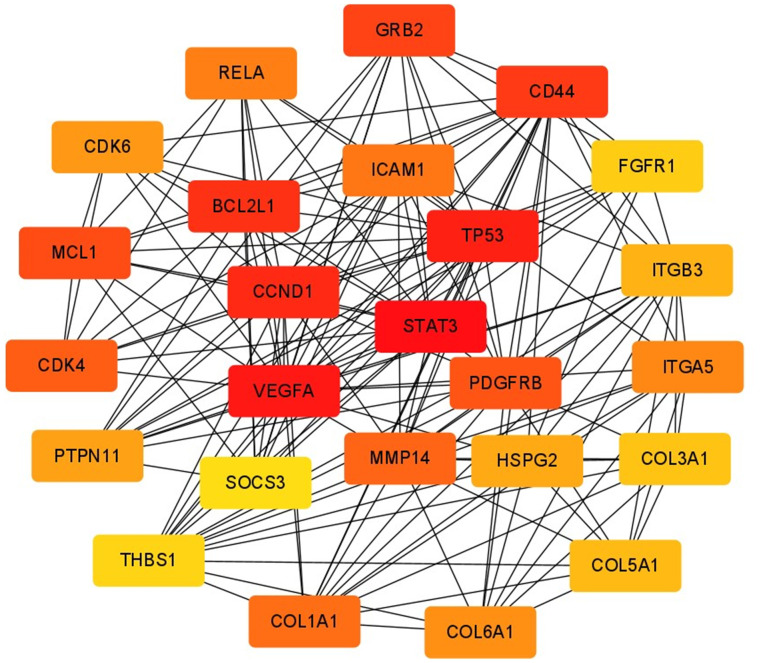
**Screening of hub gene.** The MCC algorithm in CytoHubba was used to screen hub genes. Node colors from yellow to red indicated higher and more important scores.

Among the previously predicted target genes, key regulatory genes were screened using Cytoscape’s plugin CytoHubba, and the top 25 hub genes were screened using the plugin’s maximum cluster centrality (MCC) algorithm. To calculate the clustering coefficient for each node in the network, we can use the following formula: Clustering Coefficient (C) = 2 x Number of edges between neighbors / (Degree of the node x (Degree of the node - 1)). To calculate the centrality index for each node in the network, we can use the following formula: Centrality Index (CI) = Degree of the node / (Number of nodes - 1). After calculating their clustering coefficients and centrality indices, hub genes were identified by filtering out the genes with high values in both measures. They are STAT3, VEGFA, TP53, CCND1, BCL2L1, CD44, GRB2, MCL1, PDGFRB, CDK4, MMP14, COL1A1, ICAM1, RELA, ITGA5, COL6A1, CDK6, PTPN11, HSPG2, ITGB3, COL5A1, COL3A1, FGFR1, THBS1, SOCS3, which play key regulatory roles in the target gene network (Figure 9). The score of each hub gene is shown in Table 4.

**Table 4:** Top 25 in network all string interactions short ranked by MCC method.

**Rank**	**Name**	**Score**
1	STAT3	5068502
2	VEGFA	5002339
3	TP53	4713643
4	CCND1	4214111
5	BCL2L1	2636691
6	CD44	2395251
7	GRB2	1958340
8	MCL1	1645832
9	PDGFRB	1575923
10	CDK4	1533178
11	MMP14	1421677
12	COL1A1	1409173
13	ICAM1	1179952
14	RELA	1158299
15	ITGA5	1125808
16	COL6A1	1109792
17	CDK6	1107799
18	PTPN11	1063370
19	HSPG2	1013387
20	ITGB3	996181
21	COL5A1	979841
22	COL3A1	976358
23	FGFR1	974174
24	THBS1	960846
25	SOCS3	880515

### GO enrichment and KEGG analysis of hub genes

**Figure 10:**
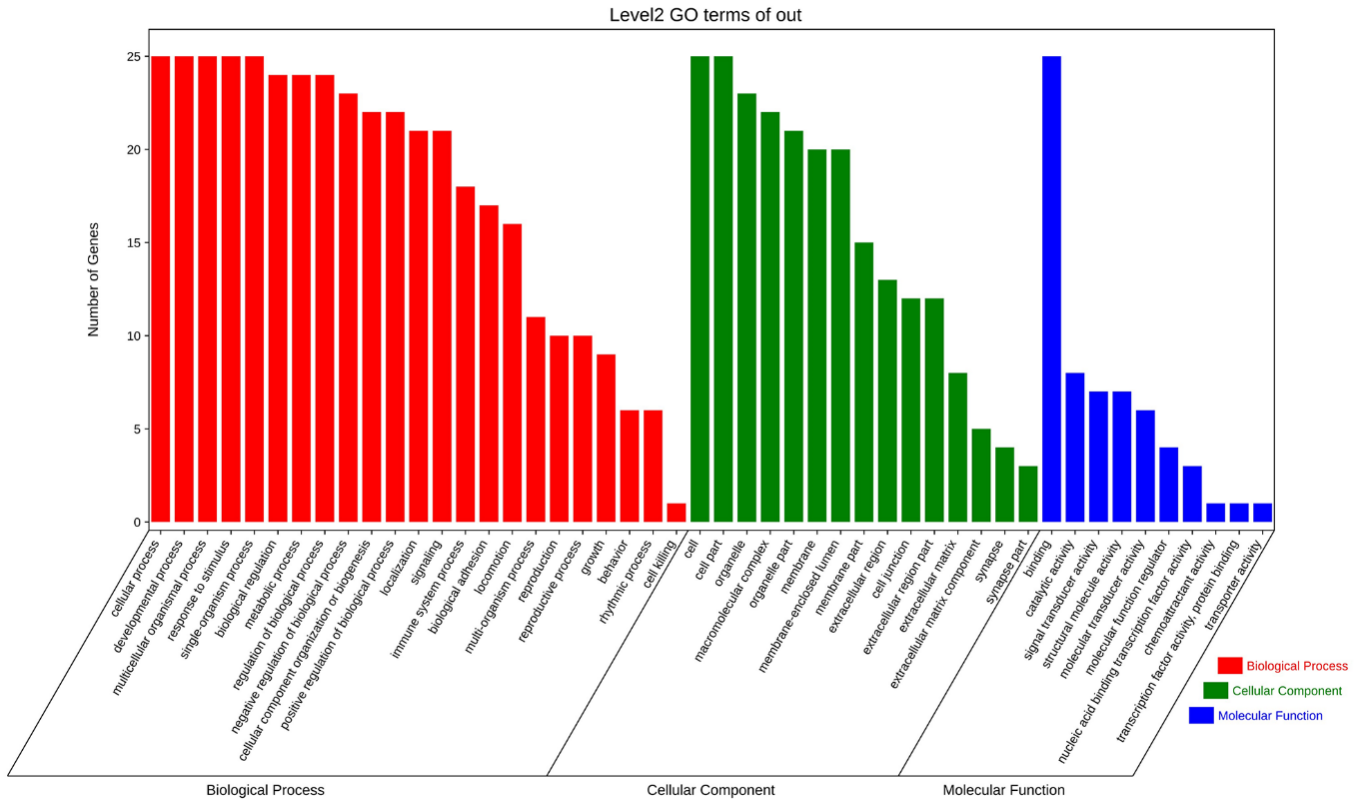
GO enrichment analysis of hub gene.

**Figure 11:**
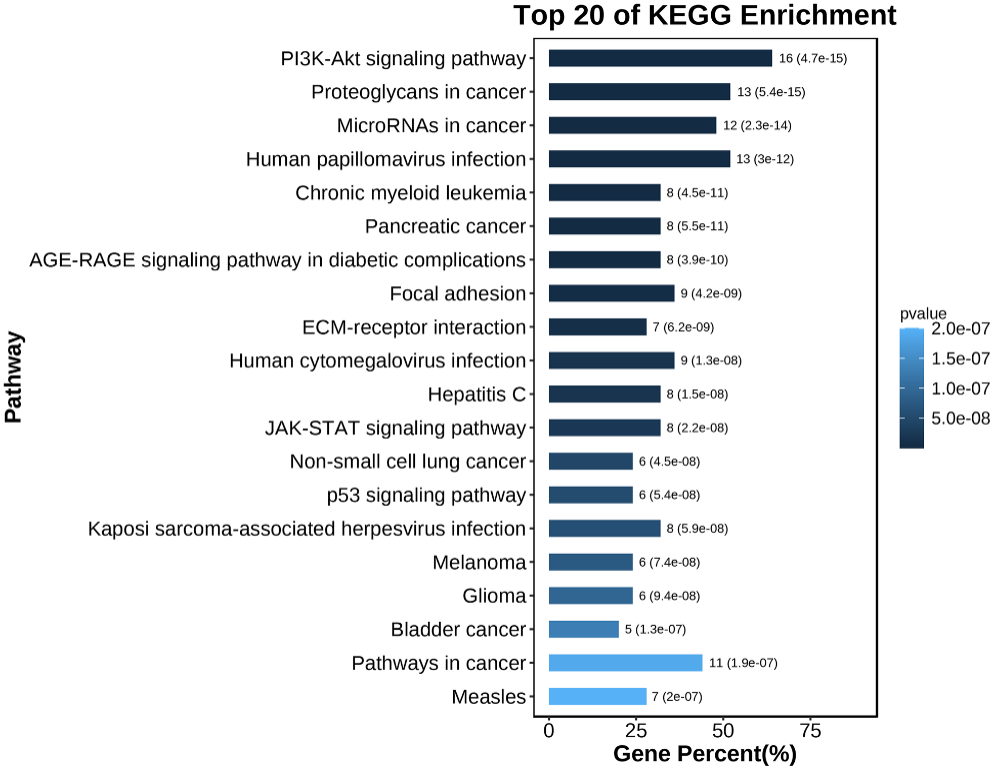
KEGG pathway enrichment analysis of the hub gene.

**Figure 12:**
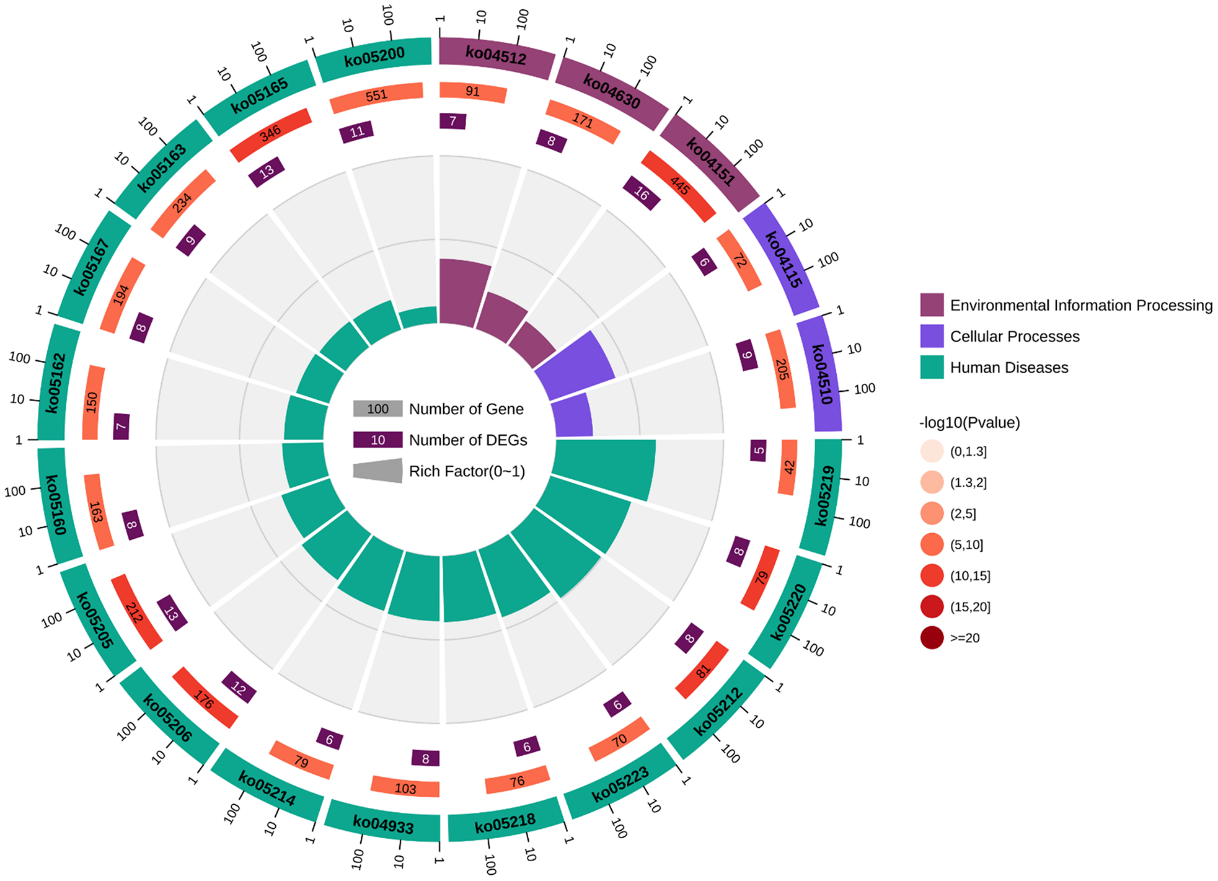
**Circular diagram illustrating the KEGG enrichment analysis.** The first circle represents the enriched KEGG IDs. The second circle represents the number of genes associated with different KEGG:ID pathways, with different colors indicating the level of gene enrichment. The third circle represents the number of genes enriched in each pathway. The fourth circle represents the proportion of genes. The darker the color of the p-value, the more significant the difference, and the change in color from light to dark indicates a transition from insignificance to significance.

To explore the biological information associated with the hub gene, we performed GO analysis and KEGG pathway enrichment using OmicShare. The results revealed that these genes were primarily involved in developmental processes, multicellular biological processes, and responses to stimuli. In terms of cellular components, the genes were predominantly located in organelles, macromolecular complexes, and membrane-enclosed lumen. Furthermore, in terms of molecular function, the genes were mainly associated with signal transducer activity and structural molecule activity (Figure 10 and Table 5). The KEGG analysis indicated that the hub gene was enriched in various pathways, including the PI3K-AKT signaling pathway, JAK-STAT signaling pathway, and others (Figures 11, 12 and Table 6).

**Table 5:** GO enrichment analysis of hub gene.

**Group**	**GO ID**	**Description**	**Out (25)**	**All (17723)**	**P-value**	**FDR**
Biological processes	GO:0032502	Developmental process	25	6569	1.63E-11	1.43E-09
	GO:0032501	Multicellular organismal process	25	7719	9.25E-10	3.60E-08
	GO:0050896	Response to stimulus	25	9222	7.95E-08	1.62E-06
	GO:0044699	Single-organism process	25	14826	0.011501	0.027604
	GO:0009987	Cellular process	25	16526	0.173872	0.198571
	GO:0050789	Regulation of biological process	24	11848	0.000565	0.002787
	GO:0008152	Metabolic process	24	12118	0.000931	0.004173
	GO:0065007	Biological regulation	24	12377	0.001484	0.00601
	GO:0048519	Negative regulation of biological process	23	5491	2.85E-10	1.34E-08
	GO:0048518	Positive regulation of biological process	22	6568	1.99E-07	3.61E-06
	GO:0071840	Cellular component organization or biogenesis	22	7131	1.06E-06	1.46E-05
	GO:0051179	Localization	21	6686	2.70E-06	3.25E-05
	GO:0023052	Signaling	21	6692	2.74E-06	3.28E-05
	GO:0002376	Immune system process	18	3279	7.82E-09	2.22E-07
	GO:0022610	Biological adhesion	17	1630	1.18E-12	1.85E-10
	GO:0040011	Locomotion	16	2010	5.30E-10	2.30E-08
	GO:0051704	Multi-organism process	11	2820	0.00082	0.003728
	GO:0022414	Reproductive process	10	1469	1.52E-05	0.000136
	GO:0000003	Reproduction	10	1472	1.55E-05	0.000138
	GO:0040007	Growth	9	945	3.16E-06	3.73E-05
	GO:0048511	Rhythmic process	6	343	6.53E-06	6.72E-05
	GO:0007610	Behavior	6	792	0.000667	0.003189
	GO:0001906	Cell killing	1	189	0.235258	0.256919
Cell components	GO:0005623	Cell	25	17034	0.115031	0.149951
	GO:0044464	Cell part	25	17034	0.115031	0.149951
	GO:0043226	Organelle	23	13845	0.028715	0.053749
	GO:0032991	Macromolecular complex	22	6212	2.44E-08	1.46E-06
	GO:0044422	Organelle part	21	10271	0.002517	0.010337
	GO:0031974	Membrane-enclosed lumen	20	5614	3.89E-07	1.02E-05
	GO:0016020	Membrane	20	9701	0.003968	0.013183
	GO:0044425	Membrane part	15	7099	0.022323	0.044754
	GO:0005576	Extracellular region	13	4295	0.00158	0.007862
	GO:0030054	Cell junction	12	1386	5.93E-08	2.60E-06
	GO:0044421	Extracellular region part	12	3465	0.000803	0.004754
	GO:0031012	Extracellular matrix	8	341	9.80E-09	1.07E-06
	GO:0044420	Extracellular matrix component	5	126	6.32E-07	1.15E-05
	GO:0045202	Synapse	4	1227	0.079211	0.117211
	GO:0044456	Synapse part	3	886	0.114435	0.149951
Molecular functions	GO:0005488	Binding	25	16807	0.103786	0.16045
	GO:0003824	Catalytic activity	8	5845	0.564888	0.600613
	GO:0004871	Signal transducer activity	7	1688	0.005911	0.024844
	GO:0005198	Structural molecule activity	7	727	3.76E-05	0.000405
	GO:0098772	Molecular function regulator	4	1450	0.129629	0.189512
	GO:0001071	Nucleic acid binding transcription factor activity	3	1416	0.301719	0.359126
	GO:0000988	Transcription factor activity, protein binding	1	522	0.513233	0.547856

**Table 6:** KEGG enrichment analysis of hub gene.

**ID**	**Description**	**Num**	**Per**	**P-value**	**Q-value**
ko04151	PI3K-Akt signaling pathway	16	64	4.69E-15	3.96E-13
ko05205	Proteoglycans in cancer	13	52	5.42E-15	3.96E-13
ko05206	MicroRNAs in cancer	12	48	2.32E-14	1.13E-12
ko05165	Human papillomavirus infection	13	52	3.04E-12	1.11E-10
ko05220	Chronic myeloid leukemia	8	32	4.45E-11	1.30E-09
ko05212	Pancreatic cancer	8	32	5.47E-11	1.33E-09
ko04933	AGE-RAGE signaling pathway in diabetic complications	8	32	3.88E-10	8.10E-09
ko04510	Focal adhesion	9	36	4.16E-09	7.60E-08
ko04512	ECM-receptor interaction	7	28	6.17E-09	1.00E-07
ko05163	Human cytomegalovirus infection	9	36	1.33E-08	1.94E-07
ko05160	Hepatitis C	8	32	1.52E-08	2.01E-07
ko04630	JAK-STAT signaling pathway	8	32	2.21E-08	2.69E-07
ko05223	Non-small cell lung cancer	6	24	4.52E-08	5.07E-07
ko04115	p53 signaling pathway	6	24	5.36E-08	5.59E-07
ko05167	Kaposi sarcoma-associated herpesvirus infection	8	32	5.93E-08	5.78E-07
ko05218	Melanoma	6	24	7.44E-08	6.79E-07
ko05214	Glioma	6	24	9.41E-08	8.08E-07
ko05219	Bladder cancer	5	20	1.28E-07	1.04E-06
ko05200	Pathways in cancer	11	44	1.90E-07	1.46E-06
ko05162	Measles	7	28	2.00E-07	1.46E-06

### Construction of miRNA and mRNA network

**Figure 13:**
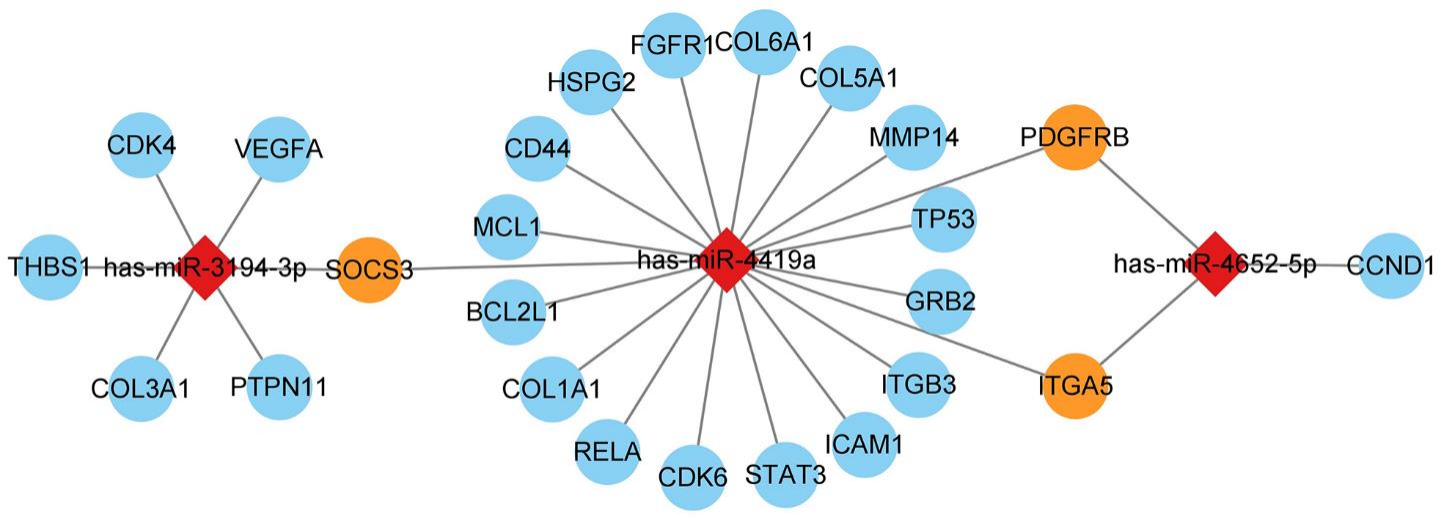
**mRNA and miRNA interact in a network, with diamonds representing miRNA and circles representing mRNA.** The blue circles represent hub genes regulated by one miRNA, while the orange circles represent hub genes regulated by two miRNAs simultaneously.

The miRNA was matched with the hub gene predicted before and was visualized by Cytoscape. Three miRNAs were matched by hub gene, namely hsa-miR-3194-3p, hsa-miR-4419a, and hsa-miR-4652-5p, all of them can regulate more than two hub genes (Figure 13).

## DISCUSSION

By analyzing serum miRNAs in healthy individuals, those with mild cognitive impairment, and Alzheimer’s disease (AD) patients, this study uncovered potential molecular mechanisms by which miRNAs regulate the development of AD through the regulation of mRNA. The study identified hsa-miR-3194-3p, hsa-miR-4652-5p, and hsa-miR-4419a as potential key players in the progression from mild cognitive impairment to AD. These miRNAs were suggested to serve as biomarkers for early AD diagnosis. Furthermore, the targeted miRNAs and central regulatory genes were predicted and analyzed to gain insights into the molecular functions of these dysregulated miRNAs in AD. GO and KEGG enrichment analysis revealed that the mRNA targets of these differentially expressed miRNAs are involved in vital biological processes such as biological adhesion, developmental processes, negative regulation of biological processes, and locomotion in multicellular organisms. These findings highlight the importance of these dysregulated miRNAs in major biological processes. Additionally, pathway enrichment analysis identified the involvement of the PI3K-AKT signaling pathway, JAK/STAT signaling pathway, and p53 signaling pathway in the pathogenesis of AD. These findings shed light on the underlying molecular mechanisms contributing to AD. Overall, this study provides valuable insights into the roles and functions of differentially expressed miRNAs in AD, unveiling their potential involvement in disease development and progression.

Dysregulation of PI3K-AKT signaling in these enrichment pathways may lead to neurodegenerative diseases by inducing molecular changes in the pathogenesis of AD. Research shows that the PI3K-AKT pathway plays a crucial role in regulating inflammatory processes in AD. Activation of this pathway can reduce the release of pro-inflammatory cytokines such as tumor necrosis factor-alpha (TNF-α) and interleukin-1 beta (IL-1β), and inhibit microglial activation and neuroinflammation [20]. Previous research has found, dysfunction of mitochondria and increased oxidative stress are commonly observed in AD brain cells, and the PI3K-AKT pathway has been shown to protect neurons by inhibiting oxidative stress and improving mitochondrial function [21]. In addition, AD pathology leads to synaptic dysfunction and impairment of memory, and the PI3K-AKT pathway plays a critical role in regulating synaptic plasticity and promoting memory formation [22]. JAK/STAT signaling pathway is emerging as a key factor in promoting neuroinflammation in neurodegenerative diseases, including Alzheimer’s disease, by initiating innate immune responses, coordinating adaptive immune mechanisms, and ultimately limiting neuroinflammatory reactions [23]. In addition, due to its involvement in receptor-mediated signal transduction activated by extracellular cytokines, the JAK/STAT pathway is implicated in cellular proliferation and differentiation, organ development, and immune homeostasis [24]. Thus, these predicted signaling pathways suggest that miRNA target genes are associated with pathological processes in AD, including neuroinflammation, neuronal apoptosis, synaptic dysfunction, and neuronal injury.

Through topological analysis of target genes, 25 central genes were obtained. STAT3, a member of the transcriptional activator family, is reported to be involved in the regulation of synaptic plasticity and cognition in hippocampal neurons [25]. STAT3 is the predicted target of hsa-miR-4419a in the current analysis. Studies have shown that overexpression of STAT3 attenuates HTAU-induced synaptic and memory dysfunction by increasing the expression of NMDAR [26]. Moreover, the phosphorylation of STAT3 in hippocampal neurons is significantly increased in mouse neurodegenerative models induced by injection of Aβ into the hippocampus. Decreased STAT3 protein attenuates Aβ-induced neuronal death [27]. Furthermore, dysfunction of STAT3 signaling is associated with Aβ formation, neuroinflammation, and increased neurotoxicity. Thus, changes in STAT3 expression represent great potential as a pathologic indicator of AD.

Other central genes may also be involved in different pathological processes of other neurodegeneration. For example, Cytokine signaling suppressor 3 (SOCS3), SOCS proteins are expressed by immune cells and central nervous system (CNS) cells and have the ability to affect the immune processes of the CNS, such as participating in the production of inflammatory cytokines and chemokines, activation of microglia, macrophages and astrocytes, immune cell infiltration and autoimmunity [28]. The expression of SOCS proteins is increased primarily by activation of the signal transductor and transcriptional activator (STAT) signaling pathways, and partly by the NF-κB pathway, both of which are induced by stimuli interacting with their receptors [29]. In the context of Alzheimer’s disease (AD), studies have explored the expression of SOCS3 in the brains of AD patients. It has been found that SOCS3 expression is significantly higher in the brains of AD patients compared to individuals with mild cognitive impairment or non-dementia individuals. Furthermore, there is a significant correlation between SOCS3 mRNA levels and the presence of Aβ plaques and neurofibrillary tangles, which are characteristic pathological features of AD [30]. This suggests that SOCS3 may play a role in AD, particularly in AD-related neuroinflammation. Interestingly, SOCS3 expression is regulated by the JAK/STAT signaling pathway, which can be activated by Aβ. This suggests a potential role for Aβ in regulating SOCS3 expression [31]. The dysregulation of SOCS3 in AD may contribute to the neuroinflammatory processes observed in the disease. By combining the analysis of various functions, the selected miRNAs and their central target genes were screened as biomarkers or potential targets directly or indirectly involved in AD.

In this study, we used healthy controls, mild cognitive impairment and Alzheimer’s disease as subjects, and obtained 5 miRNAs by qualitative blood analysis to explore the differential expression of miRNAs between patients with mild cognitive impairment and AD and healthy controls. The analysis has strong adaptability to batch effect and is suitable for individual clinical application. The differential miRNAs found in this study are expected to be an effective tool to improve the diagnostic accuracy of AD. These findings have significant clinical implications for the early detection and treatment of Alzheimer’s disease (AD). The identification of these differentially expressed microRNAs as biomarkers can enhance the accuracy of AD diagnosis and facilitate the implementation of intervention measures during the early stages of the disease. Moreover, the identification of hub genes offers potential targets for the treatment of AD. Further research is warranted to elucidate the precise roles of these hub genes in the pathogenesis of AD, which will guide the development of novel treatment strategies and medications.

In summary, the findings of this study reveal the potential application value of serum microRNAs in the early diagnosis and treatment of AD, and provide important insights for AD research and clinical practice. Further research will contribute to a deeper understanding of the role of microRNAs in the pathogenesis of AD and drive the development of personalized treatment strategies for AD.

## MATERIALS AND METHODS

### Raw data analysis

This study utilized data from the NCBI Gene Expression Omnibus (GEO) (https://www.ncbi.nlm.nih.gov/geo/) database to obtain RNA expression profiles of early cognitive impairment and Alzheimer’s disease samples. The researchers specifically screened the GSE120584 dataset, which met the necessary criteria. This dataset included 288 healthy controls, 288 Alzheimer’s disease patients, and 32 patients with mild cognitive impairment.

### Identification of differentially expressed miRNAs by GEO2R

Including the analysis of the GSE120584 microarray data sets are submitted to the online database repository GEO2R (https://www.ncbi.nlm.nih.gov/geo/geo2r/), in order to identify groups of deg. GEO2R was used for differential miRNAs analysis to obtain two groups of differential miRNAs and the Vene map was obtained by comparison. Differential miRNAs were screened with p-value ≤ 0.05, Fold change ≥ 1.5 or Fold change ≤ -1.5.

### Target gene prediction of miRNA

In order to predict the target genes that can regulate these different miRNAs, we entered these miRNAs respectively into the online database miRTarBase (https://mirtarbase.cuhk.edu.cn/~miRTarBase/miRTarBase_2022/php/index.php), RNA22 (https://cm.jefferson.edu/data-t) Database and TargetScan (https://www.targetscan.org/vert_72/). The species selected human, crossed the predicted target genes from the three databases, and drew a Venn diagram to find out what target genes were predicted jointly in the three databases.

### Analysis of proteasome interaction networks (PPIs)

The gene sets obtained from prediction site were imported into an interactive gene retrieval tool (STRING; Version 11.5; https://cn.string-db.org/) and carry out protein-protein interaction networks on the target genes, which are then imported into Cytoscape software for visualization.

### Functional annotation and pathway enrichment analysis

To gain a better understanding of the biological function of the target genes, we conducted Gene Ontology (GO) annotation and Kyoto Encyclopedia of Genes and Genomes (KEGG) pathway enrichment analyses using the OmicShare biological information cloud platform. The GO annotation included three components: biological process (BP), cellular component (CC), and molecular function (MF). Statistical significance was determined by a p-value threshold of less than 0.05. KEGG enriched and screened the top 20 items of p-value.

### Screening of key modules and identification of hub genes

Important modules were selected from the PPI network complex using the MCODE plug-in in Cytoscape. The criteria are set as degree cutoff = 2, node score cutoff = 0.2, K-core = 2, and maximum depth = 100. CytoHubba, a plugin of Cytoscape software, was used to identify the hub gene. The node with the greatest clustering centrality is selected as the important node. It can be sorted according to the calculated clustering centrality value, and the node with the largest clustering centrality value is selected as the important node.

### Data availability statement

The data that support the findings of this study are openly available in NCBI Gene Expression Omnibus database at https://www.ncbi.nlm.nih.gov/geo/, reference number GSE120584.
